# Supplementary material for: Synergistic phenotypic adaptations of motile purple sulphur bacteria Chromatium okenii during lake-to-laboratory domestication
Source: PLoS One. 2024 Oct 22;19(10):e0310265. doi: 10.1371/journal.pone.0310265 (PMC11495639; doi:10.1371/journal.pone.0310265)
Supplement: S1 File — (PDF) [file pone.0310265.s001.pdf]

Supporting Information

Phenotypic adaptations of motile purple sulphur bacteria *Chromatium okenii* during lake-to-laboratory domestication

Francesco Di Nezio[1,2], Irvine Lian Hao Ong[3], René Riedel[3], Arkajyoti Goshal[3], Jayabrata Dhar[4], Samuele Roman[1,5], Nicola Storelli[1,2], Anupam Sengupta[3*]

[1] Institute of Microbiology, Department of Environment, Constructions and Design, University of Applied Sciences and Arts of Southern Switzerland (SUPSI), Mendrisio, Switzerland

[2] Microbiology Unit, Department of Plant Sciences, University of Geneva, Geneva, Switzerland.

[3] Physics of Living Matter Group, Department of Physics and Materials Science, University of Luxembourg, Luxembourg City, Luxembourg

[4] Department of Mechanical Engineering, National Institute of Technology, Durgapur, India

[5] Alpine Biology Center Foundation, Bellinzona, Switzerland

S1 Text. Cell tracking. Cell tracking was performed using ImageJ Particle Tracker 2D/3D plug-in. Images were analysed by intensity thresholding to determine cell locations and link their position in subsequent frames, obtaining the coordinates of the cells at each interval. Cell coordinates at each frame where then used to extract single trajectories and calculate the swimming speed. Only trajectories lasting longer than 1.5 s were considered for swimming speed analysis. *C. okenii* cell body length was used as a threshold to distinguish motile from non-motile cells. For lake-sampled and laboratory-grown cells body length was set to 10 and 8 µm, respectively. Trajectories with a net displacement between 1 and 12 body lengths (10 - 120 µm) and 0.5 and 4 body lengths (4 - 32 µm) were selected for lake-sampled and laboratory cells, respectively. Cells with lower displacements were considered non-motile. Filtering was performed using custom Python code, written using NumPy library. The final filtered trajectories (*T)* were used to calculate speed at each time interval for each cell and values were averaged to obtain the mean swimming speed. Calculations were performed with custom Python code using NumPy and Pandas library. The swimming speeds (µm s^{-1}) of a population were plotted as a distribution using matplotlib module. Cells with speeds less than 1 body length were considered non-motile (*N*). To calculate the ratio of motile to non-motile cells (*R)*, total cell count (*C)* of a population (obtained by counting cells in individual frames and then averaging over all frames) was noted. The number of motile cells (*M*) was then obtained by subtracting *N* from the total number of trajectories (*T*)

$$M = T - N$$

Finally, *R* was calculated as

$$R = \frac{M}{C}$$

To highlight differences between samples in terms of motility, we arbitrarily defined three different regimes, according to cell swimming speed: no/low motility (< 5 µm s^{-1}), medium motility (5 - 20 µm s^{-1}), and high motility (> 20 µm s^{-1}).

S2 Text. Quantification of cellular mass density. The parameters used in the following calculations are:

M_{cyt}:	Mass of the cytoplasm	ρ_{cyt}:	Density of the cytoplasm	V_{cell}:	Volume of the cell
M_o:	Mass of the SGBs	ρ_o:	Density of the SGBs	V_o:	Volume of the SGBs

We define total cell mass as:

$$M_{cell} = M_{cyt} + M_o \quad (1)$$

where M_{cyt} and M_o are the mass of cytoplasm and sulfur globules, respectively. These can be expressed as:

$$M_o = V_o \cdot \rho_o$$

$$M_{cyt} = (V_{cell} - V_o)\rho_{cyt}$$

where V_o and ρ_o are the volume and density of SGBs while ρ_{cyt} is the density of the cytoplasm. Therefore, Equation (1) can be rewritten as:

$$M_{cell} = (V_{cell} - V_o)\rho_{cyt} + V_o \cdot \rho_o \quad (2)$$

which after simplification, V_o can be converted into:

$$M_{cell} = (V_{cell} \cdot \rho_{cyt}) + V_o(\rho_o - \rho_{cyt}). \quad (3)$$

Assuming SGBs to have a spherical shape and cells a spherocylindrical geometry, V_o equals $\frac{4}{3}\pi r^3$ and V_{cell} equals $\pi R^2[(h - 2R) + \frac{4}{3}R]$, Equation (3) becomes:

$$M_{cell} = (\pi R^2[(h - 2R) + \frac{4}{3}R]) \cdot \rho_{cyt} + \frac{4}{3}\pi \sum_i^n r_i^3(\rho_o - \rho_{cyt}) \quad (4)$$

where the summation indicates the sum of the volumes of the *n* SGBs inside a single cell. Dividing Equation (4) by the cell volume, the effective density of the cell can be obtained:

$$\rho_{eff} = \rho_{cyt} + \frac{\frac{4}{3}\pi \sum_i^n r_i^3 (\rho_o - \rho_{cyt})}{V_{cell}} \,. \qquad (5)$$

The fraction of the density of the cell accounted for by the SGBs is therefore represented by the term $\frac{4}{3}\pi \sum_i^n r_i^3 (\rho_o - \rho_{cyt})$.

S3 Text. Modelling mechanics. A cell generates a pusher-like propulsive force, ***P*** (because of its flagellar dynamics) to maintain its active motion. The weight of the cell (due to combined influence of the SGBs, and the rest of the cell biomass, approximated by cytoplasmic density), and the upthrust on the cell due to the finite cellular volume act in opposite directions. In addition, the cell motion induces a viscous drag (***D***, opposite to the swimming direction) that scales with the cell morphology and swimming speed. Torques on the cell structure are calculated about its centroid (or center of buoyancy, C_B, Fig 2A). The torque contributions on the cell mechanics are the following: effective torque due to the SGBs (when their effective center of mass does not coincide with C_B), the torque originating from the viscous drag (in case of asymmetric cellular geometry), and resistive (viscous) torque due to cell rotation (with rotation speed ω). Based on the physical considerations described in Fig 2a, following equations emerge from the balance of the forces and the torques:

$$P\sin\varphi = D\sin\theta$$

$$P\cos\varphi - D\cos\theta = (\rho_{cell} - \rho_{fluid})Vg = (\rho_{cyt} - \rho_{fluid})V_C g + (\rho_O - \rho_{cyt})V_O g \qquad (6)$$

$$-W\sin(\varphi)L_W = R\eta\omega$$

The symbols *ρ, V, W, η* and L_W denotes the density, volume, weight, medium viscosity, and distance from cell centroid respectively. Some of the symbols carry the subscripts *cyt, fluid, C, H, and N* which, respectively, refers to the cytoplasm, surrounding medium (within

which the cell swims), the cell, the hydrodynamic center of the cell (which coincides with the cell centroid due to its symmetrical shape), and the SGBs. Density of the cell (ρ_{cell}) is given by ρ_{fluid} times sp_{cell}, where sp_{cell} is the overall specific gravity of the cell. φ is the angle between the line of action of the propulsion force, ***P*** (originating due to the flagellar motion) and the line of action of the gravity vector. Here ω, φ and ***P*** are unknowns, which need to be determined as part of the solution. The motion of the cell does not follow the line of action of ***P***, hence an angular offset θ (an experimentally observable parameter) with the vertical direction is assumed along which the cell moves (Fig 2). φ_N is the angle between the direction of the gravity (downward, in the plane of the Fig) and the line joining C_O and C_B (note $\varphi = \varphi_N$, since we assume the center of gravity of the organelle to lie on the major axis). φ_O is the angle between the direction of gravity (vertical line) and the line joining C_O and C_B (note $\varphi = \varphi_O$, since we assume the center of gravity of the organelle to lie on the major axis). ***D*** denotes the drag force whose knowledge requires the detail of the cellular geometry and its interaction to the surrounding fluid, the details of which are provided in S3 Text.

S4 Text. Stability of swimming cells. We describe the axisymmetric cell geometry with the generic equation

$$r = \frac{ab}{\sqrt{(b^2\cos^2\gamma + a^2\sin^2\gamma)\cos^2\psi + \alpha^2 b^2\sin^2\psi}} + c\sin\psi$$

where the symbols *a, b* (*a* > *b*), $\psi(\frac{-\pi}{2} < \psi < \frac{\pi}{2})$, and $\gamma\,(0 < \gamma < 2\pi)$ represent the major axis length, minor axis length (equal to the semi-major axis length), polar angle, and azimuth angle, respectively. Here *c* implies the deviation from the symmetric shape along the major axis (fore–aft direction) and *r* denotes the position vector of the points on the cell surface (from the origin) as a function of the polar and azimuth angles. With respect to the cell geometry, *a* denotes the full length, and *b* the width of the cell.

The fore-aft asymmetry (value of *c*) is quantified using the phase-contrast microscopy images of the cells whose contours are fitted with Equation (6) and $\gamma = 0$, resulting in the form $r = \frac{ab}{\sqrt{b^2\cos^2\psi + \alpha^2\sin^2\psi}} + c\sin\psi$. Note that for a symmetric cell geometry (*c = 0*), the hydrodynamic center (C_H) falls on the cell centroid (C_B), and L_H vanishes. With the consideration that the cell shape may be assumed as a prolate spheroid, the drag of a symmetric prolate ellipsoid is expressed as $D_{\parallel,\perp} = 6\pi\eta r_{eq} U K_{\parallel,\perp}$ where *U* and *K* are the translational velocity and the shape factor, respectively, while $\parallel$ $(\perp)$ denotes the parallel (perpendicular) direction with respect to the major axis.

The shape factors have the form $K_{\parallel} = \frac{4(t^2-1)^{\frac{3}{2}}}{3t^{\frac{1}{3}}\{(2t^2-1)\ln[t+(t^2-1)^{\frac{1}{2}}]-t(t^2-1)^{\frac{1}{2}}\}}$ and $K_{\perp} = \frac{8(t^2-1)^{\frac{3}{2}}}{3t^{\frac{1}{3}}\{(2t^2-3)\ln[t+(t^2-1)^{\frac{1}{2}}]+t(t^2-1)^{\frac{1}{2}}\}}$ for prolate spheroids [34,35] where *t = a / b*. The net drag on the cell is dictated by its orientation and is given by $D = D_{\parallel}\cos(\alpha) + D_{\perp}\sin(\alpha)$ ($D_{\parallel}$ and $D_{\perp}$ are the drag forces parallel and perpendicular to the major axis of the cell shape, respectively, and $\alpha = \theta - \varphi$).

R represent the coefficient of hydrodynamic rotational resistance and has the form $R = C_R \frac{2(t^2+1)(t^2-1)^{\frac{3}{2}}}{3t\{(2t^2-1)\ln[t+(t^2-1)^{\frac{1}{2}}]-t(t^2-1)^{\frac{1}{2}}\}}$ (1) where $C_R = 8\pi r_{eq}^3$. With *R* defined, the viscous torque on a prolate spheroid is estimated using $\tau = R\eta\omega$ where ω is the angular rotation rate (rad/s). Our aim is to obtain the angular rotation rate **ω** from the above set of three coupled equations (Equation 1). Using the experimentally known values (Table 1), we draw a stability phase-plot (see Fig 3A) that enlists the value of the angular rotation rate as a function of the cell aspect ratio (*a/b*) and the ratio between the position of the cell center of weight (depending on the effective SGBs position) and the length of the long axis (L_W/a). The stability phase plots demarcate the regions of stable up-swimmers from stable down-

swimmers, thereby covering a spectrum of swimming stability conditions of *C. okenii* cells representing diverse physiological conditions.

S5 Text. Phototaxis experiment. Phototactic behaviour was observed in previously dark incubated cells after 30 min of localized LED illumination at two different light intensities (14.6 and 4.4 µmol m^{-2} s^{-1} PPFD; Fig S8). In both light regimes, *C. okenii* showed a larger ratio of highly motile cells in the illuminated sector of the millifluidic chamber, whereas in the shaded one, cells mainly fell into the low- and medium-speed regimes. In particular, the highly motile cells were substantially more when light was lower (4.4 µmol m^{-2} s^{-1} PPFD; S8B Fig), when we also observed a larger fraction of motile *vs* non motile cells (S8C Fig). Analysis of wild cell distribution revealed a significant difference in cell abundance between the illuminated and the shaded region at 4.4 µmol m^{-2} s^{-1} PPFD, while no difference was observed after exposure to higher light intensity (14.6 µmol m^{-2} s^{-1} PPFD; S8A Fig).

S6 Text. Alternative calculation of the spherocylinder aspect ratio. If we use the spherocylinder aspect ratio as the ratio between the radius of the spherical cap and the half length of the central body cylinder, the error becomes lesser. In this case, however, the total length of the cell for the spheroid and the spherocylinder does not remain the same. For spheroid the cell length is *a* (major axis) while for spherocylinder the cell length is $(a+b)/2$. In that case the error plots are shown in S4 Fig.

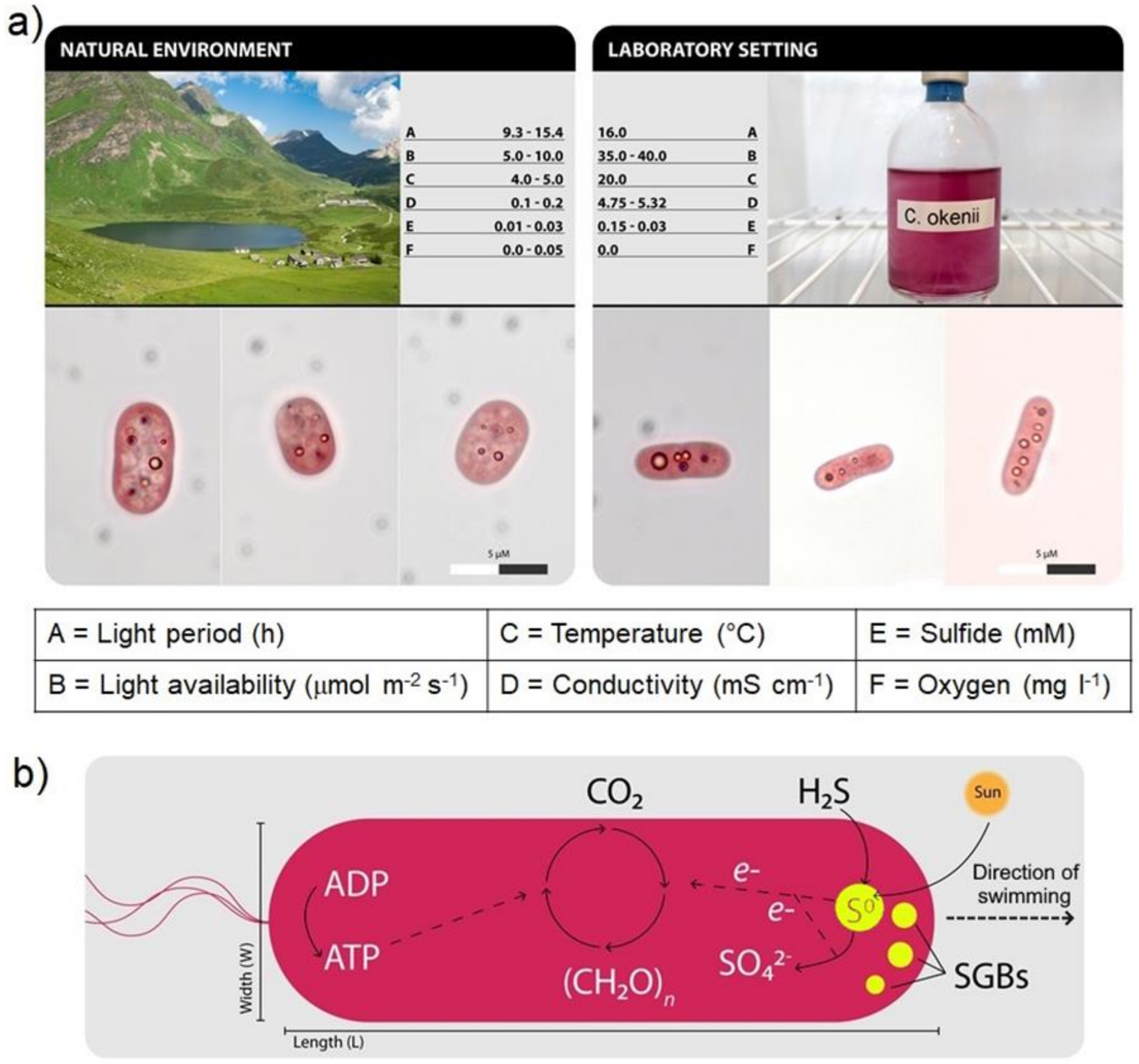

Fig S1. Difference in the physicochemical parameters measured between natural and artificial environments. a) Upper half – Different values of the main abiotic factors (A-F) influencing the growth of *C. okenii* in the natural and laboratory environment; numbers represent seasonal ranges. Lower half – light photomicrographs showing morphological differences between wild and domesticated *C. okenii* cells. **b)** Schematics of energy and reducing power synthesis in anoxygenic phototrophs. Yellow circles represent the sulfur globules inside the cells produced from the oxidation of H_2S. Length, width and SGBs number are the main features used to characterize cell morphology

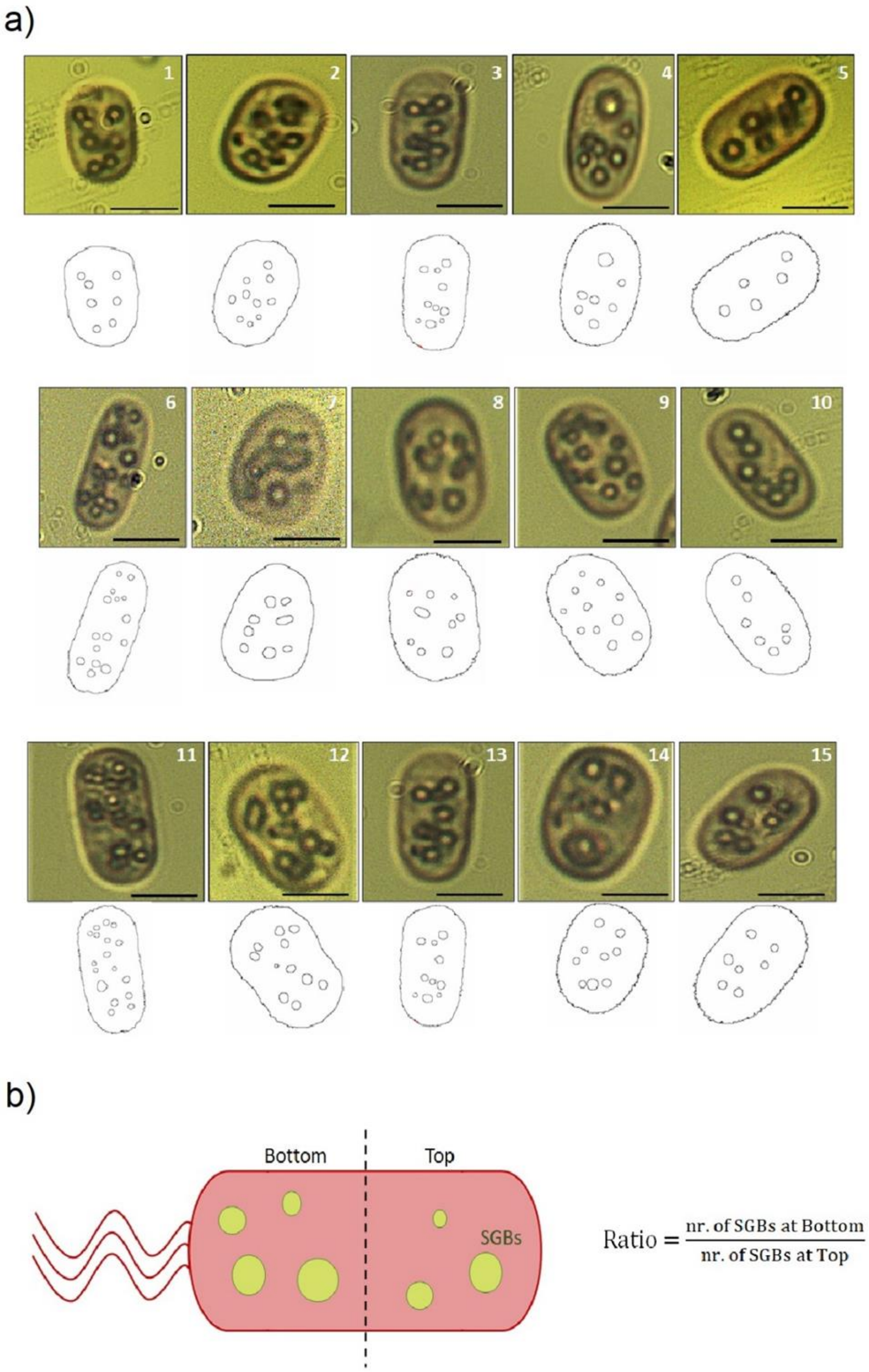

Fig S2. Determination of sulfur globules position in wild *C. okenii* cells based on single-cell microscopy. a) Top rows show micrographs obtained by light microscopy with a 100x objective (Methods) of lake-sampled cells. All micrographs are oriented so that the flagella are in the bottom part of the cell. Image analysis was used to extract the contour of each cell and the position of single sulfur globules in the cell (bottom rows). Scale bar is 5 µm. **b)** Schematic of a flagellated cell showing how the calculations were made.

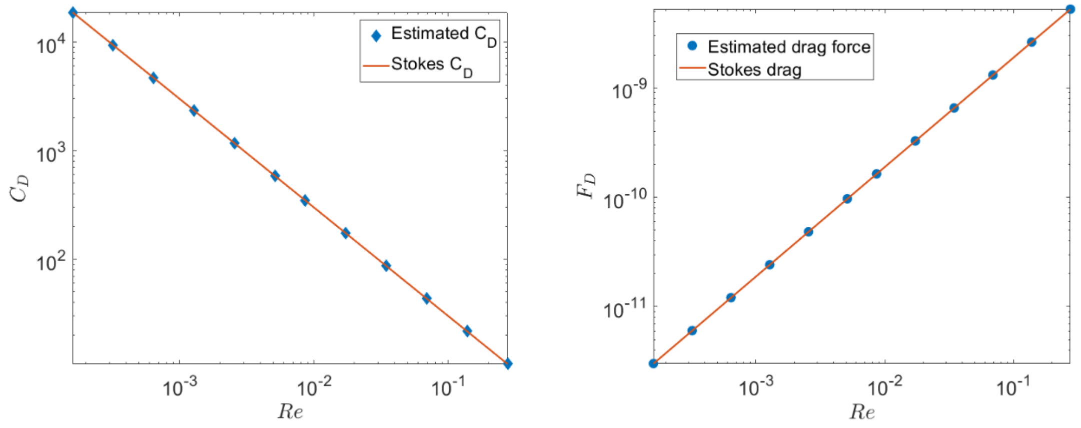

Fig S3. Validation of the COMSOL Multiphysics model for estimation of **a)** coefficient of drag and **b)** drag force for spheres.

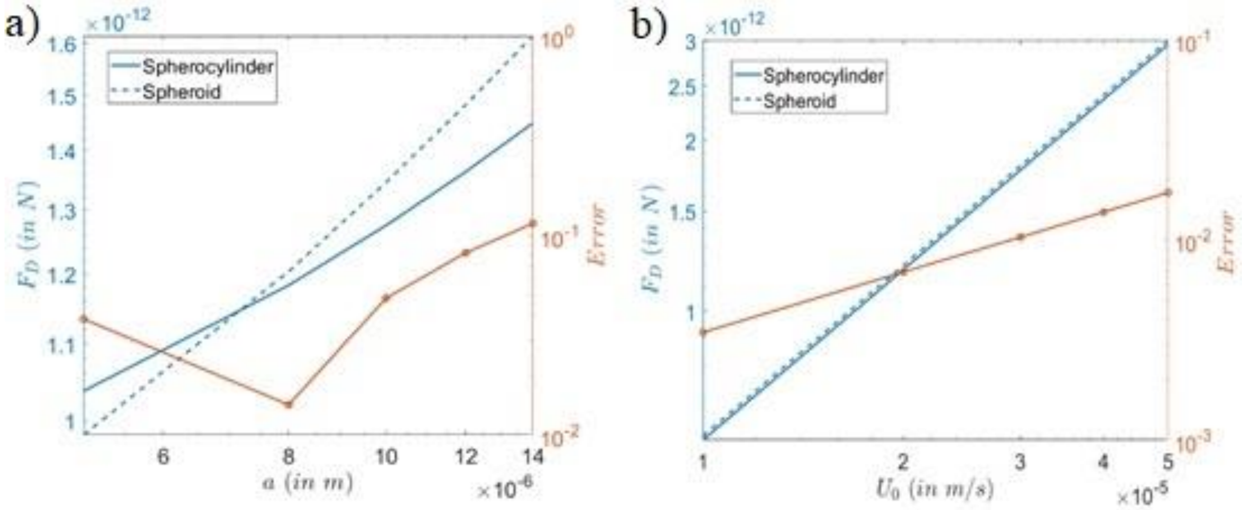

Fig S4. Comparison of drag forces between spherocylinder and spheroid cell geometries for different **a)** cell aspect ratios and **b)** swimming velocities. Here the spherocylinder aspect ratio is taken as the ratio between the radius of the spherical cap and the half length of the central body cylinder.

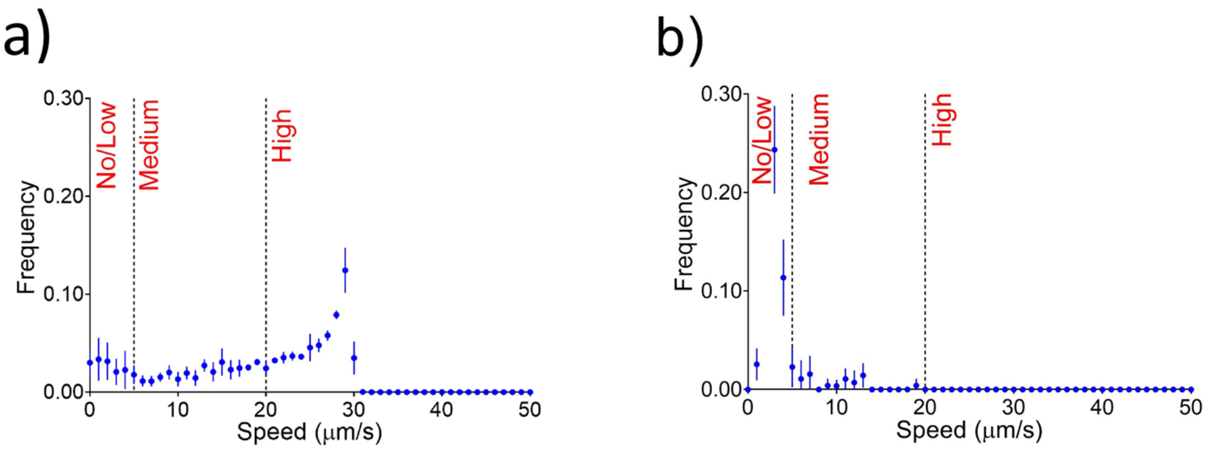

Fig S5. Histograms of the distribution of speeds of control **a)** and **b)** laboratory-grown cells in exponential phase.

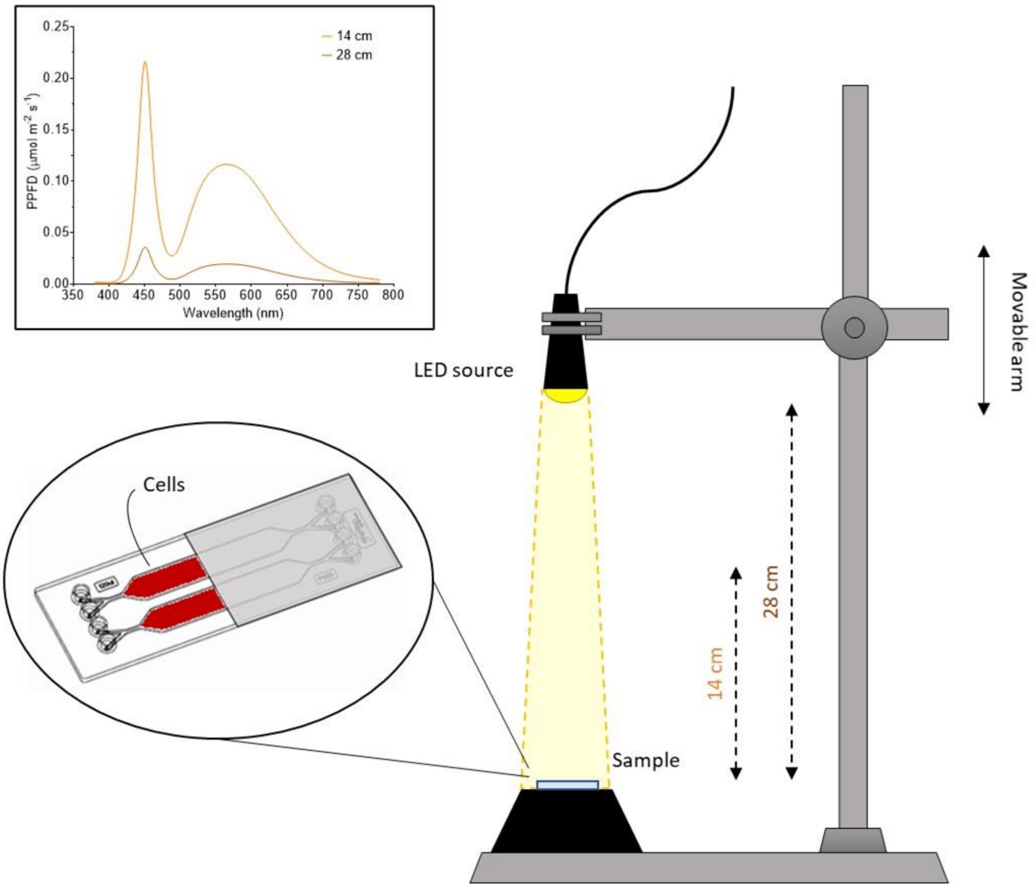

Fig S6. Schematic of the microfluidic setup. Inlet shows spectra of the LED light source at 14 and 28 cm distance from the millifluidic chip.

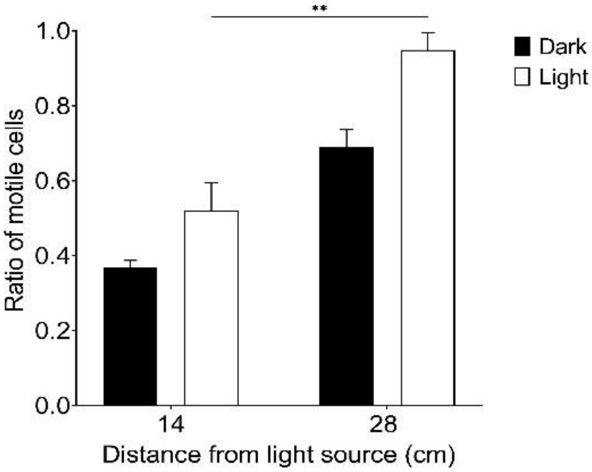

Fig S7. Different distribution of cells between dark and illuminated regions of the millifluidic device. One-way ANOVA, $p < 0.01$; post hoc Dunnet test; asterisks indicate statistically significant difference. Error bars represent standard deviation (N=3).

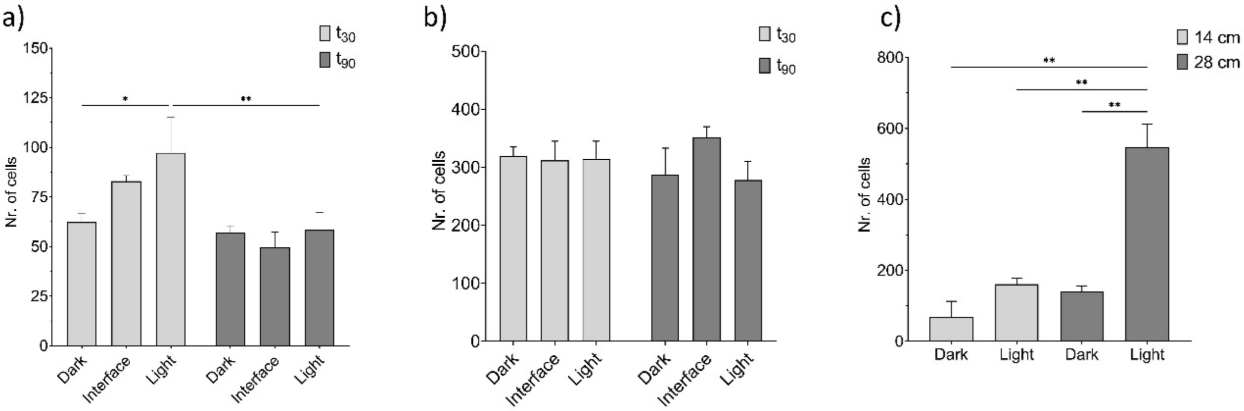

Fig S8. Distribution of cells by number in the different sections of the millifluidic device in **a)** lake-sampled and **b)** laboratory-grown cells in the half shaded-half illuminated experiment after 30 and 90 minutes. **c)** Distribution of cells by number in the dark and illuminated areas of the millifluidic device at the two different distances from the point light source. One-way ANOVA, $p < 0.01$; post hoc Dunnet test; asterisks indicate statistically significant difference. Error bars represent standard deviation (N=3).

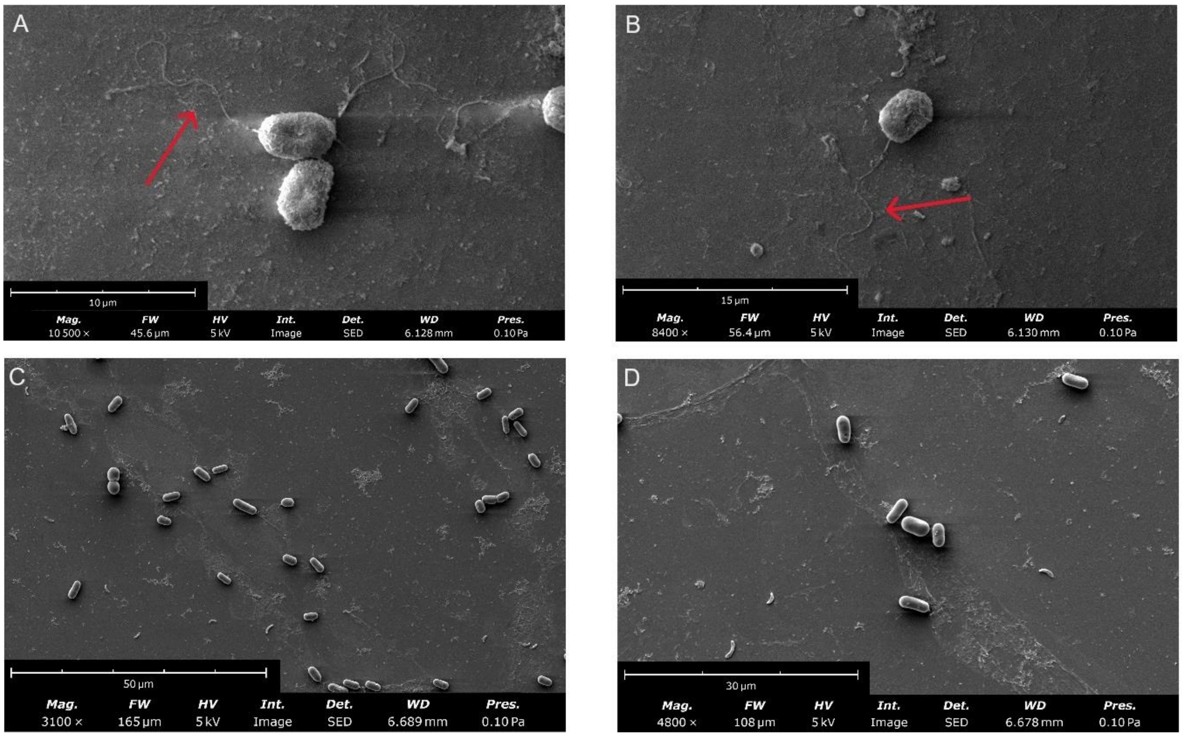

Fig S9. Scanning electron microscope images of *C. okenii* cells freshly sampled from the lake (**a,b**) and domesticated cells (**c,d**). Red arrows indicate the polar flagellar tuft.

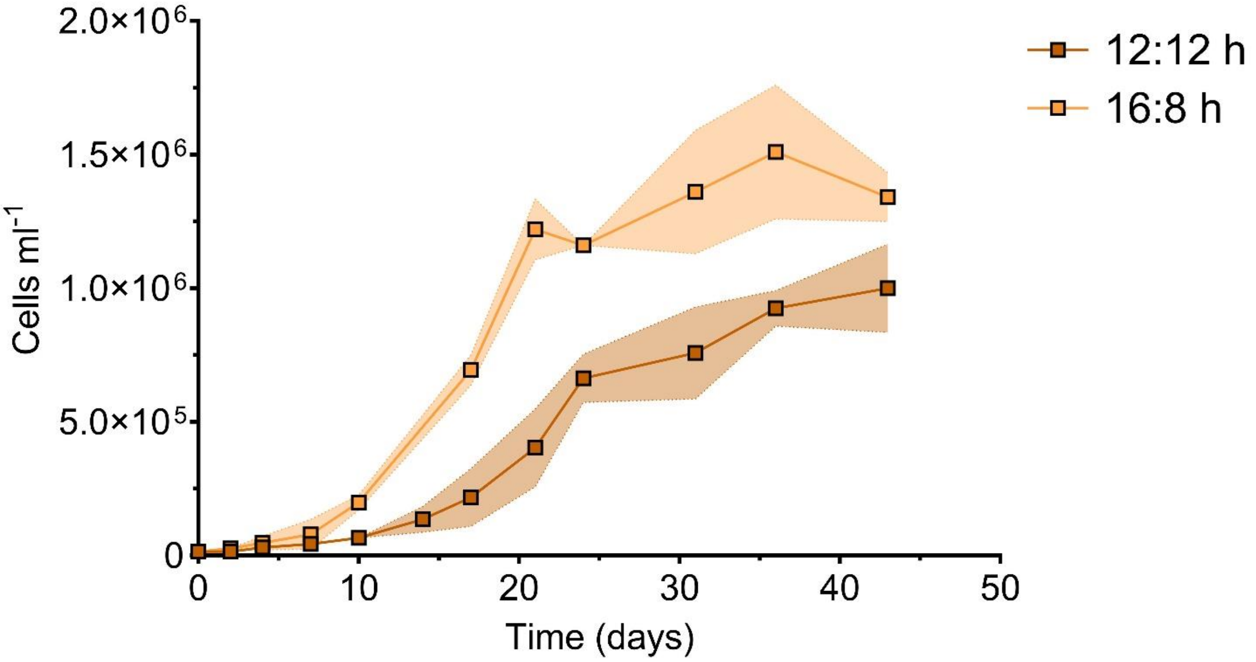

Fig S10. Different growth rates observed in *C. okenii* cells grown in the laboratory with two different light/dark photoperiods, 12/12 h (brown line) and 16/8 h (orange line).

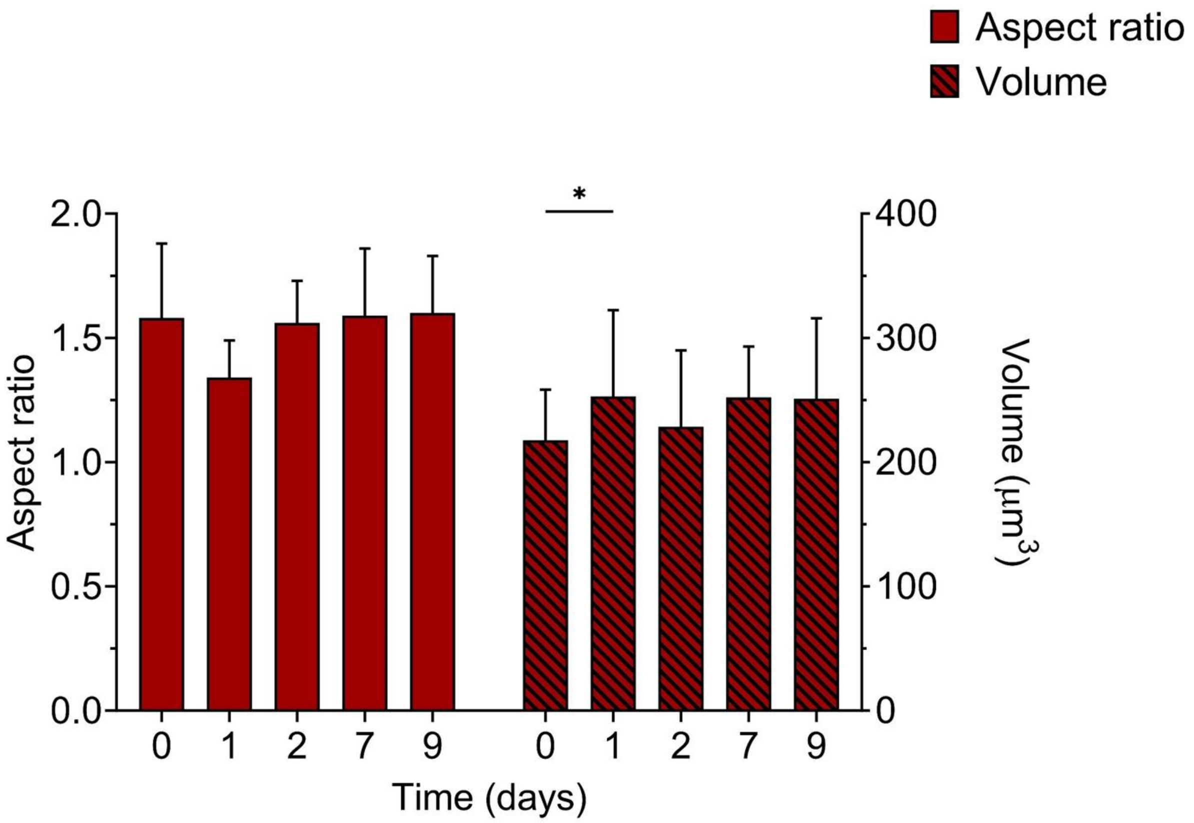

Fig S11. Wild *C. okenii* cells exhibit consistency in the main morphological traits across temperature variations (4°C to 20°C) after transition from natural to laboratory environments, as evidenced by aspect ratio and volume measurements. 2-way ANOVA, $p < 0.01$; post hoc Tukey's test. Error bars represent standard deviation (N=20).

Table S1. Parameters (µm, ± SD) used for modelling mechanics and stability of swimming cells.

	a/b	L_w	L_w/a
Lake	1.645 (± 0.292)	0.379 (± 0.270)	0.039 (± 0.025)
INC	2.231 (± 0.342)	0.412 (± 0.313)	0.079 (± 0.063)
